# Agreement on Interpretation of Point-of-Care Ultrasonography for Cardiac Tamponade Among Emergency Physicians

**DOI:** 10.7759/cureus.41913

**Published:** 2023-07-15

**Authors:** Sarah Bella, David Salo, Claire Delong, Hetal Patel, Mary Rometti, Christopher Bryczkowski, Amy Patwa

**Affiliations:** 1 Emergency Medicine, New York Presbyterian Brooklyn Methodist Hospital, New York, USA; 2 Emergency Medicine, Morristown Medical Center, Morristown, USA; 3 Emergency Medicine, Newark Beth Israel Medical Center, Newark, USA; 4 Emergency Medicine, Rutgers Robert Wood Johnson Medical School, New Brunswick, USA

**Keywords:** cardiac tamponade, pericardial effusion, tamponade physiology, emergency medicine physician, point-of-care-ultrasound, emergency echocardiography

## Abstract

Study objective: Cardiac tamponade is an impending calamitous disorder that emergency physicians need to consider and diagnose rapidly. A pericardial effusion with right atrial systolic collapse (earliest sign) or right ventricular diastolic collapse (most specific sign) and a plethoric inferior vena cava are indicators of cardiac tamponade physiology and may be identified with point-of-care ultrasonography (POCUS). The goal of this study is to assess the agreement among emergency physicians with varying levels of sonographic training and expertise in interpreting echocardiographic signs of cardiac tamponade in adult patients.

Methods: Emergency physicians at different levels of training as sonographers were surveyed at didactic conferences at three major academic medical centers in northern New Jersey. Two cardiologists were also included in the study for comparison. Survey respondents were shown 15, 20-second video clips of patients who had presented to the emergency department (ED) with or without significant pericardial effusions and were asked to rate whether tamponade physiology was present or not. Data were collected anonymously on Google Forms (Google LLC, Mountain View, CA) and included self-reported levels of POCUS expertise and level of training. Data were analyzed using Fleiss’ kappa (k). All patients had an echocardiogram performed by the department of cardiology within 24 hours of the POCUS, and the results are presented in the paper.

Results: There were 97 participant raters, including attendings, fellows, and resident physicians specializing in adult emergency medicine and two cardiologists. There was a fair degree of inter-rater agreement among all participants in interpreting whether tamponade physiology was present or not. This low level of agreement persisted across self-reported training levels and self-reported POCUS expertise, even at the expert level in both emergency medicine and cardiology specialties.

Conclusion: According to the results of our study, there appears to be a low level of agreement in the interpretation of cardiac tamponade in adult patients. The lack of agreement persisted across specialties, self-reported training levels, and self-reported ultrasonographic expertise. This low level of agreement seen among both specialists indicates that emergency physicians are not limited in their ability to determine cardiac tamponade on POCUS. This highlights the technical nature of POCUS clips and strengthens the importance of physical exam findings when diagnosing cardiac tamponade in emergency department patients. Further research utilizing POCUS for the diagnosis of tamponade is warranted.

## Introduction

Cardiac tamponade is a rare but life-threatening condition that emergency physicians need to be able to identify quickly and accurately. Pericardial effusions are seen in as many as 3.4% of individuals during autopsy for any cause of death, whereas the incidence of tamponade is 5 per 10,000 hospital admissions in the United States [1]. Emergency physicians often use point-of-care ultrasound (POCUS) in an attempt to aid in the diagnosis in conjunction with the patient's clinical presentation [2]. Clinically, cardiac tamponade is classically associated with Beck’s Triad: hypotension (weak or narrowed pulse pressure), muffled heart sounds, and jugular venous distention. However, these clinical signs are not always seen as hypotension is present early in only 48% of tamponade patients, whereas muffled heart sounds and jugular venous distension are seen in only 56% and 64%, respectively [3].

Since echocardiograms performed by the department of cardiology are not always available or may be delayed, POCUS could play a significant role in the early detection of tamponade physiology [2]. However, little is known about the level of agreement in interpreting POCUS among emergency physicians based on levels of training and expertise. This is an important consideration when developing training in POCUS for emergency medicine residents, fellows, and attendings. 

In this study, we sought to determine inter-rater reliability in the diagnosis of cardiac tamponade physiology. Emergency physicians with varying levels of sonographic expertise interpreted short clips of POCUS that had been performed in real-time and obtained from the quality assurance (QA) database. We also sought to identify aspects of cardiac POCUS that may be most helpful when evaluating tamponade physiology and could be beneficial when training residents, fellows, and attendings in using POCUS. Cardiologists' level of agreement was also included as a comparison to emergency physicians. A recent paper examined POCUS for the cardiac standstill in emergency physicians with different physician raters [4]. Our study extends the understanding of the use of POCUS for cardiac tamponade.

## Materials and methods

Study design and setting

This was a cross-sectional convenience sample survey of resident, fellow, and attending physicians in three training programs in adult emergency medicine where POCUS fellowship and training exist. The study was discussed with the Institutional Review Board (IRB) of the Atlantic Center for Research and was determined not to meet the criteria for IRB review. 

Selection of participants

Survey respondents were recruited over two months at three New Jersey emergency departments (EDs) with residencies in emergency medicine and fellowships in ultrasound with ED volumes of Hospital 1 (100,000 patients/year), Hospital 2 (95,000 patients/year), and Hospital 3 (70,000 patients/year). Volunteers were enrolled at weekly resident physician conferences and staff meetings of attending physicians. Conference participants were attending physicians, residents, and fellows in ultrasound, who are all in the specialty of adult emergency medicine. Respondents were allowed to participate only once and asked not to discuss the project until the data collection was completed. Data was not collected from or tracked for conference attendees who elected not to participate. Two cardiologists from Hospital 1 were randomly chosen and also asked to review the same POCUS images.

Methods of measurement

During study enrollment, a basic definition of cardiac tamponade along with the sonographic features of cardiac tamponade were discussed. Cardiac tamponade physiology was defined as compression of the heart by fluid in the pericardial sac, and ultrasonographic features reviewed included significant pericardial effusion, diastolic collapse of the right ventricle (most specific sign), systolic right atrial collapse (earliest sign), and a plethoric inferior vena cava (IVC) with minimal respiratory variation. Rater demographics were collected, including current training level (resident, fellow, or attending), self-reported POCUS skill level (non-user, basic, advanced, or expert), and institution of practice. Clinicians who agreed to participate were given access to a quick response (QR) code linked to a Google Form (Google LLC, Mountain View, CA, USA) survey where answers were electronically collected. Participants used personal cellular devices to answer survey questions. No identifying information was collected on the participants other than their training level, reported POCUS skill, and institution. 

After demographic information was obtained, participants were shown 15 clinical scenarios associated with a subsequent cardiac POCUS video clip. The POCUS clips for these cases had been collected using phased array probes on the Mindray M9 (Mindray Medical Int. Ltd., Shenzhen, CHN) and Zonare Z.One PRO (Mindray Medical Int. Ltd.) ultrasound systems on patients who had presented to the Hospital 1 ED within the past two years. The POCUS in these videos was performed by emergency physicians during the period of patient evaluation and included resident physicians, fellows, and attendings who had received formal training in POCUS. These video clips were entered into the Hospital 1 ED QA database. 

During testing, clinical scenario slides (see Appendix 1) that included a brief history of the present illness and vital signs were shown prior to each video clip. The deidentified video clips were 6 seconds in length and looped for a total of 20 seconds for the participants to review. During the 20-second videos, the raters were asked to answer the question, "Does this image suggest cardiac tamponade physiology?" with the choice to answer 'yes', 'no', or 'unable to determine'. After the 20-second time frame, polling was closed and the next slide was presented. The time frame of 20 seconds was chosen to simulate a previous study that assessed the variability in interpreting cardiac standstill [4]. The 15 clips selected included a range of cardiac views such as parasternal long, parasternal short, subxiphoid, and apical views in patients with pericardial effusions to demonstrate the wide variability seen in emergency medicine clinical practice. These clips were chosen to analyze the agreement of the responses from the participants. A transthoracic echocardiogram performed by the department of cardiology was performed on all patients within 24 hours of presentation to the ED, and the results of these echocardiograms were presented as the gold standard. To compare further image interpretation by emergency physicians to that of cardiologists, two cardiologists from Hospital 1 were asked to review the same images with subsequent recordings of their level of agreement. Additionally, the cardiologists were asked based on a Likert scale to rate the quality of the POCUS that were shown in the study, with the lowest score of 1 meaning strong disagreement, a median score of 3 meaning neutral agreement, and the highest score of 5 meaning strong agreement. 

Outcome measures

The primary outcome measure was the level of agreement in the interpretation of cardiac tamponade physiology among all respondents compared to the gold standard transthoracic echocardiogram. Secondary outcomes measured included the level of agreement among the subgroups of training level, self-described POCUS experience level, and institution. Data were further examined to determine POCUS views with the greatest and least agreement to help determine if certain POCUS views appeared to be more helpful at the bedside for the diagnosis of tamponade physiology.

Primary data analysis

We assessed inter-rater reliability among emergency physician sonographers and by subgroup, using Fleiss’ coefficient. Data from the Google Forms survey were exported to a Microsoft Excel database (Microsoft Corp., Redmond, WA, USA) and analyzed with SPSS Statistics version 28 (IBM Corp., Armonk, NY, USA). 

## Results

Characteristics of study subjects

We surveyed 95 emergency physician sonographers composed of attendings, fellows, and residents from three major academic institutions in northern New Jersey and two cardiologists. The most represented training level was resident (52%), self-reported POCUS skill level was basic (74%), and the institution was Hospital 3 (41%). Comprehensive demographics are listed in Table 1.

**Table 1:** Demographics and agreement among respondents POCUS: Point-of-care ultrasonography

Characteristics	N	%	Kappa	Agreement
Training Level				
Attending	38	40%	0.15	Poor
Resident	49	52%	0.27	Fair
Fellow	8	8%	0.23	Fair
Self-reported POCUS skill level				
None	4	4%	0.02	Poor
Basic	70	74%	0.23	Fair
Advanced	17	18%	0.21	Fair
Expert	4	4%	0.26	Fair
Institution				
Hospital 1	36	37%	0.22	Fair
Hospital 2	20	22%	0.24	Fair
Hospital 3	39	41%	0.20	Poor
Cardiologists	2	2%	0.25	Fair

No exclusions were noted when performing the subgroup analysis as participants were required to answer all survey questions. Of the respondents who reported no POCUS experience, all four identified themselves as attending physicians. Although we cannot be certain, these participants may have completed residency training prior to the advent of POCUS in the field of emergency medicine or have not had enough experience with POCUS to consider themselves at a basic skill level. 

Main results

**Figure 1:**
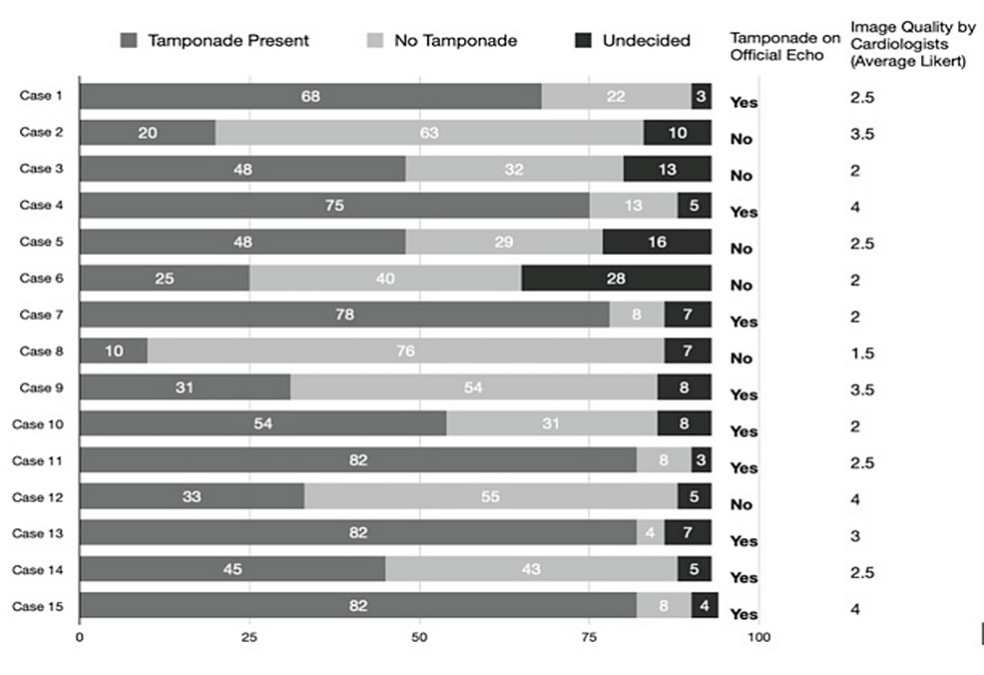
All survey responses

Among all 97 participants, there was only a fair level of inter-rater agreement (kappa (k)=0.21). Table 1 shows kappa values broken down by training level, self-reported POCUS skill level, institution, and specialty, with all kappa results indicating fair or poor agreement among respondents. Figure 1 demonstrates the variability in agreement across the 15 POCUS clips. Certain clips, such as 4, 11, and 13 resulted in a stronger agreement for cardiac tamponade. While clips 3, 5, 6, 9, and 12 may have had subtler findings of tamponade physiology and resulted in larger percentages of respondents favoring contradictory interpretations.

Regardless of specialty, training level, or POCUS experience, there was a low degree of inter-rater agreement among all participants. Among subgroups, residents had the highest agreement (k=0.27) while attending physicians and self-reported skill as "none" had the lowest agreement (k=0.02). Our response rate was 100% for the entire cohort. Respondents were unable to decide whether a clip was tamponade physiology or not within the 20-second time limit for 129 of the 1396 possible responses. The average Likert score of each image quality rated by the two cardiologists appears on the right side of Figure 1. Cases 2, 4, 9, 12, and 15 had higher Likert scale ratings (3.5 or greater) while cases 3, 6, 7, 8, and 10 had lower Likert scale scores (2.5 or less). Interestingly, both emergency physicians and cardiologists found case 4 highly agreeable, and case 6 had a lower agreement for cardiac tamponade physiology.

## Discussion

In this cross-sectional study, performed at three New Jersey EDs located at academic medical centers, emergency physicians with varying levels of POCUS training had a low inter-rater agreement ranging from 0.02 to 0.27 when interpreting cardiac tamponade physiology as viewed from POCUS videos. A previous study with a similar design demonstrated mid- to low-level agreement for cardiac standstill (k=0.40-0.55) [4]. In that study, emergency physicians likewise had varying levels of POCUS training. Though overall agreement in their study was slightly better than in ours (k=0.47 vs. k=0.21), the agreement was lower than expected in both studies. This raises several questions: 1) is the self-reported POCUS skill level responsible for the low level of agreement? Or 2), is the technical nature of POCUS for tamponade physiology the issue? In this study, 74% of respondents classified themselves as having basic ultrasound knowledge, which could make it more difficult for them to determine tamponade physiology and therefore dramatically impact the overall level of agreement. However, since the level of agreement in the cardiac standstill study [4] was fairly constant among emergency physicians with varying levels of training, i.e., those who completed fellowship training vs. those with minimal training, it would appear that the quality of POCUS images shown may have been the limiting factor. Arguably, performing POCUS for a tamponade would be more difficult than for a cardiac standstill. This is because multiple views are necessary with the ability to pause and apply advanced POCUS applications, making quality images challenging to obtain. Since our study showed a narrow, low range of agreement between those with little training and fellowship-trained physicians, this supports the idea that the technical nature of POCUS images is responsible for low levels of agreement. Although POCUS identification of cardiac tamponade can help improve patient outcomes and expedite care, it requires expertise to be accurately performed. 

The POCUS is vital in confirming cardiac tamponade physiology when clinical suspicion is high [2,5,6]. One of the key features is the presence of a pericardial effusion, which can best be seen in parasternal long and subxiphoid views [4]. Besides the presence of a pericardial effusion, other key components viewed on the POCUS include the diastolic collapse of the right heart and vena cava enlargement [5,7,8]. Right ventricle diastolic collapse is specific (75% to 90%) for cardiac tamponade and not as sensitive (48% to 60%), while right atrial systolic collapse is sensitive (50% in early tamponade to 100%) [5,7]. Right atrial systolic collapse is one of the earliest ultrasonographic signs of cardiac tamponade [9]. A dilated IVC is very sensitive (95% to 97%) for cardiac tamponade, but lacks specificity (~40%) [5,7]. Despite numerous cardiac POCUS features indicating cardiac tamponade physiology, the diagnosis is also a clinical one. Findings on POCUS can be paired with hemodynamic instability in making the diagnosis [5,7,10-12]. Rapid diagnosis of cardiac tamponade by POCUS can expedite treatment such as emergent pericardiocentesis and decrease hospital length of stay [10,11,13,14]. A strength of this study was including real-time patient images from the Hospital 1 ED to realistically reflect clinical practice.

The cardiologist agreement data were added to this study to compare their level of agreement with emergency physicians and to rate the quality of the images shown. The cardiologist, similar to emergency physicians, had only fair agreement on image interpretation as shown in Table 1. Again, this may reflect an issue with the quality of images obtained by POCUS shown in this study rather than an ability to interpret cardiac tamponade. In this study, only one cardiac view was displayed per case and only for 20 seconds. Participants were not able to pause, repeat, or apply advanced sonographic features to the image for better evaluation. This differs drastically from performing the POCUS image in real time where multiple cardiac views, the ability to pause the image, apply motion (M)-mode, and evaluate the IVC can be obtained. The Likert scale scores from the two cardiologists would further support this idea as many images were rated as low quality (a score of 2.5 or less on the Likert scale). This again highlights the complexity of diagnosing cardiac tamponade physiology and the need for physicians to also consider the patient's clinical context. 

There are several limitations to this study. First, this study is a cross-sectional sample of video clips that were performed by physicians with varying levels of training. This variable could account for the variation in image quality when compared to images obtained by emergency physicians with fellowship ultrasound training. Second, the selection of study participants may not have captured all available emergency physicians from each of the three major academic institutions and could represent sample bias since only those presenting at resident conferences and faculty meetings were enrolled. Although efforts were made to make the viewing of the presentation similar among the three medical centers, inevitable differences in the setting such as room lighting, projector size, or room size, could have influenced participants viewing of the images. Study participants were asked not to speak during the presentation; however, audience discussion may have occurred that could influence responses. Overall, we do not believe that there was a significant degradation in image quality and audience discussion. Third, we relied on self-reported levels of expertise, similar to a previous study [4]. While this could account for low kappa levels, we again emphasize that low kappa levels existed among all expertise levels. Pre-testing to determine levels of ultrasound training or formal guidelines on what would constitute different levels of expertise might help control for variances in self-reported levels in future studies. These guidelines would help control for residents who may have more or less training for their postgraduate year (PGY) level due to previous ultrasound experience. Fourth, 20-second clips may not have been adequate time for participants to determine if tamponade physiology was present. A longer clip duration along with multiple cardiac views may have impacted agreement and study design. 

The images shown to the audience included a range of different cardiac views including parasternal long, parasternal short, apical view, and subxiphoid view, to reflect the wide variety of images seen in our daily POCUS practice. Only one cardiac view was included in the clip shown for each patient, which could make interpretation difficult as the chosen image shown in the study may not have been the optimal image to determine tamponade physiology. For clips with less agreement, the right ventricle may have been difficult to visualize, no significant pericardial effusion was noted, and right atrial collapse, an early sign of tamponade physiology but potentially a less recognized sign, may have been seen. Clips with better agreement had clear views of right ventricular diastolic collapse with obvious pericardial effusions. There were a few "unable to determine" responses in cases with better agreement than in cases with worse agreement, suggesting that there may be less provider confidence when more difficult cases were interpreted. An additional limitation would be not including a view of the IVC, as it typically would be plethoric in the setting of cardiac tamponade, and performing a sniff test to help aid in determining if tamponade physiology is present. Also, having the ability to slow the image down and apply M-mode to determine the cardiac phase could be helpful. Images were only shown for 20 seconds, which differs from performing POCUS at a patient's bedside where a longer period would be permitted to view and interpret the image for tamponade physiology. The participants may be more familiar with looking for right ventricular diastolic collapse instead of subtler findings such as right atrial systolic collapse that will be present in early cardiac tamponade. Lastly, cardiac tamponade may be a dynamic process, and the echocardiogram performed by the department of cardiology used as a gold standard in this study could have differed from the images that were obtained at the bedside during the ED course. 

## Conclusions

In conclusion, our results support a low degree of inter-rater agreement among all participants in determining cardiac tamponade physiology, even at an expert level that includes cardiologists. The physicians that were included in this study had widely varying interpretations of cardiac tamponade physiology. Among our results, clips that included clear views of right diastolic collapse had more agreement vs. other clips that included subtler findings such as right systolic atrial collapse or views where the right ventricle may not have been clear, leading to more disagreement. To our knowledge, no study to date has reviewed the level of agreement among emergency physicians in determining cardiac tamponade physiology. The risk of misinterpreting cardiac tamponade could be devastating to patients and delay definitive treatment. This low level of agreement seen among both specialists indicates that emergency physicians are not limited in their ability to determine cardiac tamponade on POCUS. This highlights the technical nature of POCUS clips and strengthens the importance of considering multiple cardiac views in conjunction with physical exam findings when diagnosing cardiac tamponade in ED patients. Future studies of cardiac tamponade may further investigate the POCUS features of cardiac tamponade.

## Appendices

Appendix A 

**Table 2:** Clinical scenarios including a brief history of the present illness and vital signs Slides with the above details were shown to participants prior to each corresponding video clip. HR: Heart rate, BP: Blood pressure, SOB: Shortness of breath

Scenario	Age	Sex	HR (BPM)	BP (mmHg)	Temperature	Oxygen saturation	Respiratory rate	Chief complaint
1	53	F	105	137/104	Afebrile	96% room air	20	Worsening SOB with minimal exertion x 3 to 5 days
2	61	M	121	122/88	Afebrile	96% 2 L nasal cannula	18	Exertional SOB and fatigue x 1 week
3	75	F	123	137/55	Afebrile	95% room air	18	Chest pain x 2 weeks
4	58	M	56	68/52	Afebrile	92% room air	15	Generalized weakness and dizziness
5	77	M	93	119/52	Afebrile	99% room air	18	SOB x 3 weeks with recent COVID-19 infection
6	28	M	88	138/99	Afebrile	99% 4 L nasal cannula	36	Chest pain and SOB x 1 day
7	61	F	99	115/84	Afebrile	93% 4 L nasal cannula	20	Progressive SOB x 3 to 4 weeks
8	55	F	109	101/73	Afebrile	97% room air	20	Pleuritic chest pain x 3 days
9	57	F	115	135/68	Afebrile	96% room air	22	Few weeks of SOB
10	92	F	76	124/62	Afebrile	97% room air	18	SOB with recent pacemaker placement
11	48	F	130	99/83	Afebrile	93% 2 L nasal cannula	20	SOB x 2 days
12	80	F	107	175/87	Afebrile	96% 15 L non-rebreather mask	18	Worsening SOB over the last few days
13	83	M	62	81/57	Afebrile	100% room air	18	Worsening SOB over the last few days
14	65	M	97	163/110	Afebrile	88% room air	24	SOB and hypoxia to 50 to 80s on room air at home
15	62	F	71	126/85	Afebrile	99% room air	21	Intermittent, dry cough x 2 to 3 weeks
